# The Circadian Rhythm of the Behavior and Gut Microbiota in Dybowski’s Frogs (*Rana dybowskii*) during the Autumn Migration Period

**DOI:** 10.3390/life14030322

**Published:** 2024-02-28

**Authors:** Nan Hu, Yingdong Li, Meizhang Wang, Haoyu Ji, Xian Zhang, Baolong San, Hongyue Shi

**Affiliations:** College of Animal Science and Veterinary Medicine, Shenyang Agricultural University, Shenyang 110866, China

**Keywords:** circadian rhythm, *Rana dybowskii*, behavior, gut microbiota, amphibians

## Abstract

Many amphibian behaviors and physiological functions adapt to daily environmental changes through variations in circadian rhythms. However, these adaptations have yet to be reported in Dybowski’s frog (*Rana dybowskii*). We aimed to elucidate the dynamic changes in the behavior and gut microbiota of *R. dybowskii* within a 24 h cycle during their migration to hibernation sites. Thus, we monitored their behavior at 4 h intervals and collected samples for microbiome analysis. We found that the juvenile frogs arrived at hibernation sites earlier than the adults. Among the adults, the male frogs arrived earlier. The richness and diversity of the gut microbiota in the adult *R. dybowskii* were lowest at 14:00. At 6:00, the differences between the males and females were most significant. At 18:00, there was an increase in the activity of *Bacteroides*, *Coprobacillus*, *Ruminococcus*, and *Dorea* in the intestinal tracts of the male frogs, whereas in the intestinal tract of the female frogs, there was an increase in the activity of *Pseudoramibacter_Eubacterium*, *Desulfovibrio*, *Anaerotruncus*, and *PW3*. This indicated diurnal rhythmic variations in the gut microbiota and significant sex-based differences in the microbial activity at different time points. Our findings contribute to the understanding of the circadian rhythm of *R. dybowskii* and provide crucial insights into improving breeding strategies.

## 1. Introduction

Circadian rhythms have long been a notable focus in the field of animal biology. The circadian cycle not only influences animal behavior [1] but also affects physiological processes, such as metabolism and hormone secretion [2]. Previous studies on circadian rhythms have primarily focused on mammals [3] and birds [4]. However, studies on the circadian rhythms of amphibians have revealed the orcadian activity of *Rana kl. Esculenta* [5] and plasma corticosterone in *Bufo americanus* [6]. The circadian rhythm changes in response to environmental factors, such as light [7] and temperature [8]. In our previous studies [9,10], we revealed the circadian rhythm of Dybowski’s frog (*Rana dybowskii*) and the impact of photoperiod on its Melatonin. These studies have revealed the importance of circadian rhythms in maintaining homeostasis and adapting to the environment. However, research on the circadian rhythm of *R. dybowskii* is limited.

Animal behavior is an important direction of biological research and a direct reflection of animal responses to environmental changes, mainly concentrated in birds and mammals [11,12]. In recent years, research on the reproductive habits, life history, and other aspects of amphibians has gradually been reported. Lamoureux provided a detailed description of the seasonal behavior of green frogs [13], while Michel et al. explored the impact of the developmental environment on amphibian behavior [14]. In addition, there have been reports on behaviors, such as vocalizations, courtship, and antipredator behavior [15,16,17]. In summary, these preliminary behavioral studies provide a necessary foundation for understanding amphibians and provide certain reference value for the protection and rational development of some endangered or vulnerable species with high economic value. However, because these studies only used basic descriptions, it is difficult to understand the essence of animal behavior at a deeper level. For this reason, many scholars have conducted in-depth research on certain behaviors of amphibians from physiological and molecular ecology perspectives [18,19]. For example, Woodley reviewed the relationship between chemical signals, hormones, and amphibian reproduction [20].

The gut microbiota also play an important role in host physiological processes, including metabolism [21], immunity [22], and adaptability [23]. Additionally, factors such as the environment, diet, and life history can affect the structure and function of the gut microbiome. In *Rana sphenocephala*, the gut microbiota of tadpoles is influenced by diet composition [24]. In green frog (*Lithobates clamitans*) tadpoles, both environmental temperature and microbial communities influence relative brain mass and shape [25]. Research on Asiatic toads (*Bufo gargarizans*) has revealed considerable structural differences between the large and small intestines [26]. Moreover, rotten-skin disease is shown to significantly alter the gut microbiota of the giant spiny frog (*Paa spinosa*) [27]. As a variable temperature vertebrate, the larvae (tadpoles) of frogs live in water, while the majority of adults live on land. They have characteristics that fall between fish and reptiles. This lifestyle also creates its unique gut microbiota. According to reports, the gut microbiota of tadpoles is similar to that of fish [28], while adult frogs are more similar to mammals than fish [22]. This depends on their lifestyle and environmental factors. In recent years, research on the gut microbiota of frogs has gradually deepened, but there have been no reports on its circadian rhythm changes.

*Rana dybowskii*, an amphibian found in Northeast China, has substantial economic and ecological value locally [29]. In recent years, the population of *R. dybowskii* has decreased [30]. However, studies on this species are limited. As an important stage of life for *R. dybowskii*, migration is a preparation for hibernation and breeding. If not successfully completed, it will directly affect the survival rate. Therefore, we aimed to investigate the circadian rhythms of *R. dybowskii* behavior and gut microbiota during the autumn migration by observing its behavior at different feeding times and analyzing the abundance and diversity of its gut microbes as it transitioned from terrestrial to underwater hibernation. The results of this study provide a valuable reference for enhancing the breeding efficiency of *R. dybowskii*, particularly in light of its unique characteristics.

## 2. Materials and Methods

### 2.1. Ethics Statement

This research was conducted with species that are neither endangered nor protected. All the experiments were carried out in accordance with the guidelines for scientific purposes, animal care, and use formulated by the Animal Ethics Committee of Shenyang Agricultural University, Approval Code: 2023081801 and Approval Date: 2023-08-20. All possible measures were taken to minimize discomfort and distress to the animals involved.

### 2.2. Sample Collection

On 29 September 2023, *R. dybowskii* frogs were collected from a natural forest and hibernation site within the breeding facility in Dandong City, Liaoning Province, China. Three sets of interception points were established in the forest near the hibernation site, each with a length of 150 m. Observations were recorded every 4 h during a 24 h period on a single day (2:00, 6:00, 10:00, 14:00, 18:00, and 22:00 local time). General data regarding the number, age, sex, length, and weight of the frogs were recorded using visual observations, calipers, and an electronic scale. Nets were placed across the bottom of the pond to count the number of frogs arriving at the eastern hibernation site. The frogs along the shore were then manually counted and collected from throughout the pond to determine their total number in the water. Simultaneously, three adult male and three adult female frogs were collected at each time interval, and we immediately euthanized the frogs in the wild by severing the spinal cord. The intestinal specimens were preserved in liquid nitrogen and dispatched to Shanghai Personal Biotechnology Co., Ltd. (Shanghai, China) for DNA extraction and Illumina sequencing. All the samples were collected under natural light during the day or dim red light at night.

### 2.3. DNA Extraction

Genomic DNA from the samples was isolated using the OMEGA Soil DNA Kit (M5635-02) provided by Omega Bio-Tek in Norcross, GA, USA, adhering to the guidelines specified by the manufacturer, and was then preserved at −20 °C awaiting further examination. The quantity and quality of the extracted DNA were measured using a NanoDrop NC2000 spectrophotometer (Thermo Fisher Scientific, Waltham, MA, USA) and agarose gel electrophoresis, respectively.

### 2.4. 16S rRNA Gene Amplicon Sequencing

The V3–V4 region of the bacterial 16S rRNA gene was amplified using polymerase chain reaction (PCR) with the forward primer 799F (5′-ACTCCTACGGGAGGCAGCA-3′) and reverse primer 1193R (5′-TCGGACTACHVGGGTWTCTAAT-3′). The PCR reaction mix included 1 μL of the DNA template. The process was carried out in an Applied Biosystems 2720 thermal cycler (Invert Logan, Carlsbad, CA, USA), following a specific thermal cycling protocol: an initial denaturation step at 98 °C for 5 min; 25 cycles of denaturation at 98 °C for 30 s, annealing at 53 °C for 30 s, and extension at 72 °C for 45 s; and concluding with a final extension step at 72 °C for 5 min.

### 2.5. Bioinformatics and Statistical Analysis

The analysis of the microbiome’s bioinformatics was carried out with QIIME2 version 2019.4, adhering closely to the protocol outlined in the official guide available at https://docs.qiime2.org/2019.4/tutorials/ (accessed on 30 October 2023), with slight adjustments. The initial processing of the raw sequencing data involved demultiplexing through the q2-demux plugin and subsequent primer removal utilizing the q2-cutadapt plugin [31]. The sequencing reads underwent filtration, noise reduction, merging, and the elimination of chimeras, all achieved through the application of the DADA2 plugin [32].

A sequence data analysis was performed using QIIME2 and R package version 3.2.0. Alpha diversity (including Chao1, observed species, Shannon, and Simpson indices) was computed at the ASV level with QIIME2’s ASV table and presented through box plots. The ASV abundance and diversity across samples were compared using abundance curves. For beta diversity, which examines variations in microbial communities among samples, we used Bray–Curtis dissimilarity metrics and visualized the results via PCoA, NMDS, and hierarchical clustering [33]. Using the “Venn Diagram” R package, a Venn diagram was generated to show the shared and unique ASVs in a sample or population based on the occurrence of ASVs in different samples and populations, regardless of their relative abundance [34].

The behavioral data are presented as the mean ± SD. The statistical comparisons were conducted using one-way ANOVA, with Duncan’s test identifying significant mean differences at *p* < 0.05. SPSS version 26.0 was used for all the statistical analyses. The images were created with Microsoft Excel 2021 and CorelDRAW Graphics Suite 2022.

## 3. Results

### 3.1. Basic Metrics of R. dybowskii

In total, 225 froglets and 176 adult frogs were detected (Table 1). The male-to-female ratio was approximately 1:2. There were no significant differences in the body length or weight among the froglets. The average weight of the female adult frogs was 24.24 g, with an average body length of 62.41 mm, which were significantly greater than the average weight and length of the male frogs (*p* < 0.05).

### 3.2. Circadian Rhythms in the Behavior of R. dybowskii

**Figure 1:**
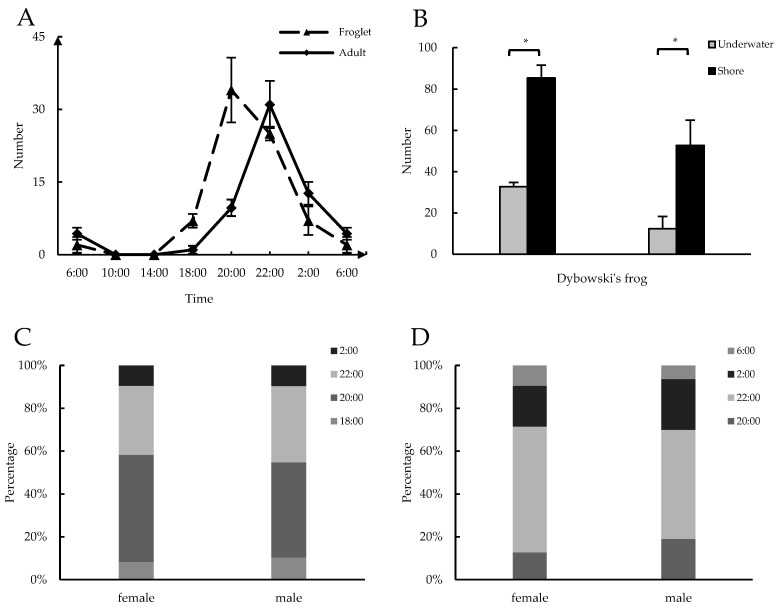
(**A**) Migration rhythms of *R. dybowskii*. Each time point represents the mean (*n* = 3, mean ± SD). (**B**) The behavior of *R. dybowskii* upon reaching the hibernation sites, with each set of data representing the mean (*n* = 3, mean ± SD). (**C**) The comparison of the sex differences in the primary time periods of migration occurrence (Froglet). (**D**) The comparison of the sex differences in the primary time periods of migration occurrence (adult frog). Asterisks indicate statistically significant differences between pairs of values (* *p* < 0.05).

In the 24 h observation results, the migratory behavior primarily occurred in the evening and early at night. The peak migration period for the froglets was 20:00, whereas that for the adult frogs was 22:00 (Figure 1A). There was no sex-based difference in the migratory behavior of the froglets (Figure 1C); however, the adult male frogs arrived at the interception points earlier than the adult females (Figure 1D). Additionally, as shown in Figure 1B, significantly more frogs near the hibernation site stayed on the shore during the daytime compared to the number entering the water (*p* < 0.05).

### 3.3. Bacterial Sequencing of the Gut Microbiota

In total, 2,864,357 original sequences were read. After quality control, denoising, and chimera removal, 1,908,366 sequences remained (female: 869,907; and male: 1,038,459). We identified 86 bacteria at the phylum level, 6198 at the genus level, and 1635 at the species level (Tables S1 and S2).

### 3.4. Alpha Diversity of the Gut Microbial Community

**Figure 2:**
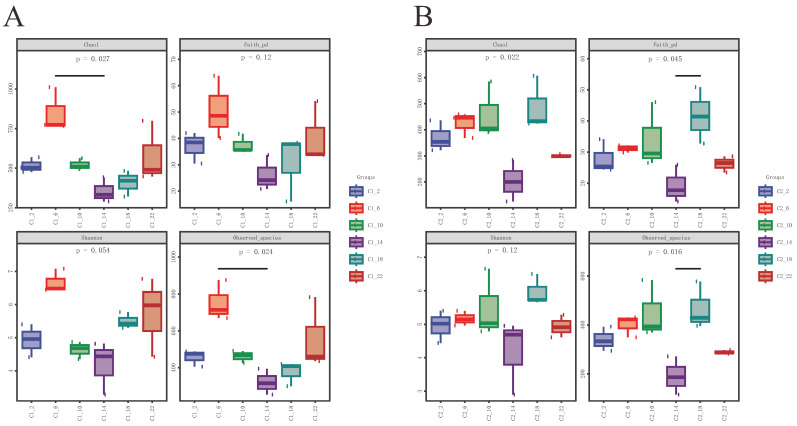
Alpha diversity indices (Chao1, Faith_pd, Shannon, and observed species) of the gut bacterial community at six time points over a day and night. Box plots depict the medians (central horizontal lines), inter-quartile ranges (boxes), and 95% confidence intervals (whiskers). *p*-values are from the Kruskal–Wallis test. Asterisks indicate statistically significant differences between pairs of values (* *p* < 0.05). (**A**) Male. (**B**) Female.

**Figure 3:**
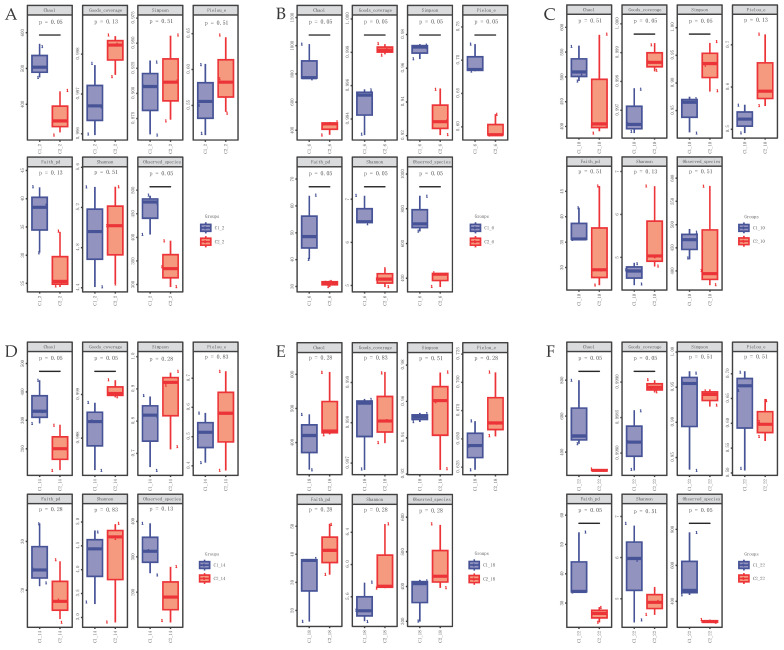
Sex differences in the alpha diversity indices of the gut bacterial community at the same time point. Box plots depict the medians (central horizontal lines), inter-quartile ranges (boxes), and 95% confidence intervals (whiskers). *p*-values are from the Kruskal–Wallis test. Asterisks indicate statistically significant differences between pairs of values (*p* < 0.05). (**A**) 2:00. (**B**) 6:00. (**C**) 10:00. (**D**) 14:00. (**E**) 18:00. (**F**) 22:00.

Six time points were compared using the alpha diversity indices to assess the richness and diversity of the gut microbiota. In the male frogs, the Chao1 index and number of observed species were significantly higher at 6:00 than at 14:00 (Figure 2A), whereas in the female frogs, the Faith_pd and number of observed species were significantly higher at 18:00 than at 14:00 (Figure 2B). No significant differences were observed in the Shannon index. As shown in Figure 3, there were significant sex-based variations in the diversity and richness of the gut microbiota at most time points. In particular, the richness and diversity of the microbial community showed significant differences in all the indices at 6:00 (Figure 3B), indicating that sex-based differences were most pronounced at this time. However, there were no significant sex-based differences in the alpha diversity indices at 18:00 (Figure 3E).

### 3.5. Analysis of Microbial Community Structure

**Figure 4:**
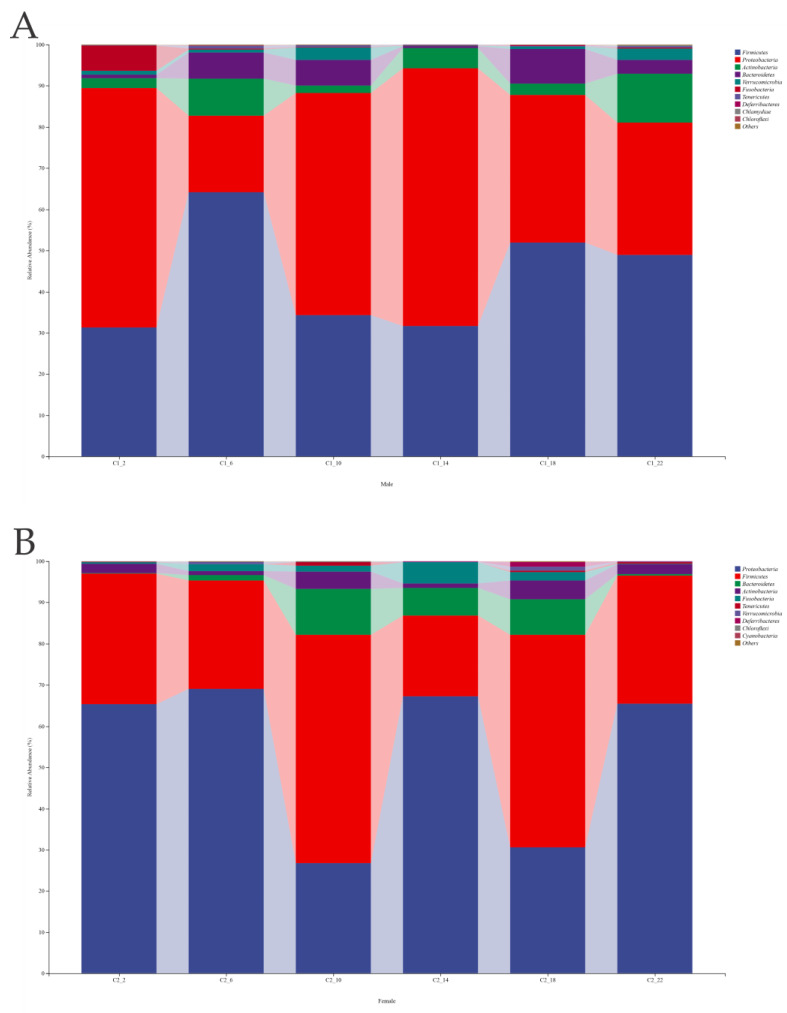
The gut bacterial community’s relative abundance at the phylum level. (**A**) Male. (**B**) Female.

The three main phyla (>1% relative abundance) found in the intestinal samples of the male frogs were *Firmicutes*, *Proteobacteria*, and *Actinobacteria* (Figure 4A), whereas the three main phyla in the female frogs were *Proteobacteria*, *Firmicutes*, and *Bacteroidetes* (Figure 4B). These dominant phyla collectively accounted for over 90% of the total relative abundance of the gut microbiota. Among the dominant phyla in the male frogs, *Firmicutes* had the highest proportion at 6:00 (64.12%), whereas *Proteobacteria* had the lowest proportion (18.62%). Meanwhile, *Proteobacteria* had the highest proportion at 14:00 (62.53%). In the female frogs, *Proteobacteria* constituted the highest proportion at 6:00 (69.08%), whereas the relative abundance of *Bacteroidetes* was lower at night (2:00, 0.2%; and 22:00, 0.3%).

The most prevalent genus was *Serratia*; however, the abundance of other genera varied throughout the 24 h observation period. In the male frogs (Figure S1A), *Carnobacterium* exhibited its highest relative abundance at 6:00, while in the female frogs, *Carnobacterium* reached its lowest relative abundance at 14:00 (Figure S1B). At the species level, *Cetobacterium somerae* abundance in the male frogs was significantly higher at 2:00 compared with that at other time points (Figure S1C). In the female frogs, *Eubacterium dolichum* had the highest relative abundance at 18:00 (Figure S1D).

Based on the microbial community detection in the samples, there were significant differences in the quantity of intestinal bacteria in *R. dybowskii* at different time points (Figure S2). The quantity in the male frogs at different times was ranked from low to high as follows: 14:00 (270), 18:00 (464), 2:00 (501), 10:00 (618), 22:00 (741), and 6:00 (1036). For the female frogs, the ranking was as follows: 14:00 (251), 22:00 (317), 2:00 (349), 6:00 (442), 10:00 (614), and 18:00 (758).

**Figure 5:**
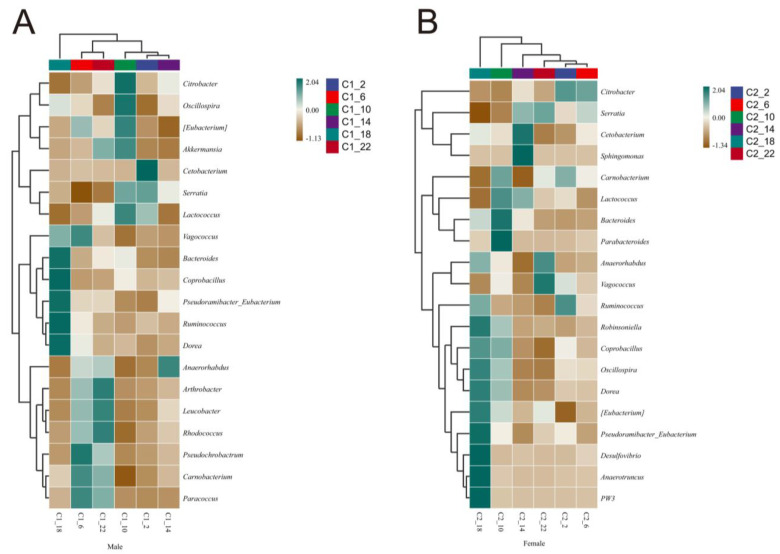
Correlation analyses of the gut bacteria. (**A**) Male. (**B**) Female.

The species composition heatmap showed the presence of circadian rhythms in the intestinal microbial community of *R. dybowskii*, with notable differences between the males and females. At the phylum level (Figure S3A), the abundances of *Chlamydiae*, *Gemmatimonadetes*, *Synergistetes*, and *NC10* were higher in the male frogs at 22:00. At the genus level (Figure 5A), *Bacteroides*, *Coprobacillus*, *Pseudoramibacter_Eubacterium*, *Ruminococcus*, and *Dorea* showed higher at 18:00 than at other times. Notably, *Cetobacterium*, which contributes to digestion and absorption, had a significantly higher relative abundance at 2:00 compared to that at other times. However, the female frogs had a higher abundance at 14:00, and similar results were observed at the species level. In the female frogs (Figure S3D), *Akkermansia muciniphila* were higher at 6:00 than at other times or higher than that of the males, whereas *Prevotella ruminicola*, *Bacteroides uniformis*, and *Bacteroides fragilis*, which contribute to digestion and absorption, were more abundant at 14:00.

### 3.6. Beta Diversity and Sexual Dimorphism in R. dybowskii

Beta diversity is also referred to as between-habitat diversity, and in this study, the microbiomes of frogs serve as habitats for bacteria. The beta diversity index (Bray–Curtis dissimilarity) was obtained using PCoA and NMDS (Figure S4), and there were significant differences between the microbial communities of the males and females.

### 3.7. Analysis of Metabolic Pathways

We determined the abundance of the active metabolic pathways in the microbiome by consulting various metabolic pathway databases and applying calculation methods (Figure S5). The metabolism pathway category contained the most enriched pathways, including carbohydrate metabolism, amino acid metabolism, cofactor and vitamin metabolism, and terpenoid and polyketide metabolism.

## 4. Discussion

The field of ethology holds paramount importance in biology, as it focuses on direct responses to external environmental stimuli [35]. Migration behavior, a common phenomenon in the natural world [36,37], has received limited attention in amphibian research. This study marks an inaugural investigation of the circadian rhythms of behavior in *R. dybowskii*. The findings indicate a distinctive behavioral pattern regarding migration. Unlike avian species that migrate during the daylight hours [38], many species of amphibians migrate at night during periods of rain [39,40]. The migration behavior of *R. dybowskii* occurs from late September to early October, and it begins when the temperature reaches below 10 °C during rainy autumn nights and is primarily concentrated in the late evening and early nocturnal hours. The underlying factors contributing to these results may be attributed to humid conditions on rainy days, which ensure adequate skin moisture and prevent surface contamination by soil and debris, thereby facilitating faster movement. As a poikilothermic organism, frogs reduce their activity in response to the decline in temperature after 22:00, and this serves as a primary factor in the observed reduction in frog abundance during the latter part of the night. This behavior distinguishes *R. dybowskii* from *Rana temporaria* [41], which can complete its migration during the day due to the relatively higher temperature and humidity levels in its habitat.

In our research, we found that the froglets arrived at the hibernation sites earlier than the adult frogs. This may be because froglets are smaller and have weaker mobility, which keeps them closer to water sources during the forest-dwelling stage. Similar age-related differences in migration have been observed in *Ambystoma maculatum* [39]. Subsequently, a statistical analysis of 183 froglets at the hibernation site revealed that the majority congregated at the shoreline and only entered the water for refuge when startled. Once frogs permanently enter the water, they no longer feed until six months later [42]. This is because foraging only occurs on land. This indicates that increased foraging toward the end of the season may be essential for building lipid reserves in preparation for hibernation [43]. These findings offer new insights into the migratory behavior of amphibians.

The gut microbiota is crucial in maintaining the host’s internal balance and plays a vital role in growth and development [44]. An organism’s gut microbiome is a key element in maintaining its daily rhythm [45]. Among the hydrobionts, there is a significant degree of variation in the dominant members of the gut microbial community. For example, fish communities are rich in *Proteobacteria* [46], while amphibious communities are dominated by *Firmicutes* and *Bacteroidetes* [47]. In the present study, *Firmicutes* and *Proteobacteria* were identified as the keystone phyla of the gut microbiome, whereas the phyla *Bacteroidetes* and *Actinobacteria* were also abundant. This is consistent with the abundances found in frogs such as *Babina adenopleura* [48], *Polypedates megacephalus* [49], and *Odorrana tormota* [50], suggesting that *Firmicutes*, *Proteobacteria*, and *Bacteroidetes* are the dominant phyla in the amphibian gut microbiota.

The results of the alpha diversity analysis showed that the abundance and diversity of the bacteria identified in the gut microbiome varied significantly throughout the day. At 6:00, the microbial community in the intestinal tract of the male frogs was significantly larger than that at 14:00, whereas in the female frogs, it was significantly larger at 18:00 than at 14:00. This indicated that the gut microbiota of *R. dybowskii* exhibited the lowest richness and diversity at 14:00, which corresponds to a vacuum period of digestion and absorption in frogs. These results suggest the influence of the circadian rhythm on gut microbial composition and abundance. However, temperature fluctuations can also affect the frog’s gut microbiota [51]. During the migration period, there is a large temperature difference between the day and night, resulting in poor gut microbiota richness and diversity at 14:00. Furthermore, the impact of habitat changes in amphibians is equally important [52,53]. Frogs migrate from the forest to live by the pond, and this process, such as soil microorganisms, predation, and competition, can cause changes in its gut microbiota. A lack of diversity in the gut microbiota may result in an elevated stress response and a weakened immune system [54,55]. Similar results have been reported in other frog species [29,56], and our research confirms these findings.

Amphibians typically display sexual dimorphism [57], and we found significant differences between the microbial communities of male and female *R. dybowskii.* A species composition heatmap revealed increased activity of *Bacteroides*, *Coprobacillus*, *Pseudoramibacter_Eubacterium*, *Ruminococcus*, and *Dorea* at 18:00 in the male frogs, which are associated with functions such as decomposition, absorption, and maintenance of the immune system [58,59,60]. In the female frogs, there was increased activity of *Desulfovibrio* and *Anaerotruncus*, which promote fat accumulation. This is likely due to the need of females to store a significant amount of fat for gonadal development at this time [61]. In contrast, studies on *Fejervarya limnocharis* [62] and *Bufo gargarizans* [63] did not find the gut microbiota to be influenced by sex. Amphibians may also display sexual dimorphism in terms of size [64]. For example, in *R. dybowskii*, females are significantly larger than males. Previous studies demonstrated that frogs of various sizes exhibited different prey preferences [65]. Furthermore, it is widely recognized that diet is a key factor influencing gut microbiota composition and metabolism [66]. Therefore, slight differences in the types of food consumed by males and females in the same region may lead to differences in the gut microbiota. It is important to note that other factors, such as hormonal microbes, may also contribute to these differences.

The gut microbiota of many animals undergoes seasonal changes, mainly due to their adaptation to the environment [67]. The *R. dybowskii* is in the transition stage from terrestrial life to underwater hibernation during migration, so we speculated that its gut microbiota will affect digestion and metabolic functions to a certain extent. Therefore, we conducted metabolic pathway statistics and discovered many pathways involved in carbohydrate, amino acid, and lipid metabolism. As is well known, the food of frogs is rich in chitin, and chitinase is produced in the stomach. We have evidence to suggest that the gut may be an important site for the further breakdown of chitin. This is consistent with Li’s findings [62], further supporting our results. This may give the *R. dybowskii* better digestive ability. In addition, the lipid metabolism ability of frogs is enhanced, allowing them to obtain sufficient energy during the hibernation period when nutrients are lacking. Research has shown that in *R. dybowskii*, the ratio of *Firmicutes* to *Bacteroidetes* decreased from 4.23 in the summer to 0.63 in the winter to adapt to the hibernation stage [42]. In summary, the gut microbiota of *R. dybowskii* plays an important role in regulating the body’s nutrition during migration.

## 5. Conclusions

The peak migration period of *R. dybowskii* occurs in the evening, with juvenile frogs reaching hibernation sites relatively early, possibly due to their proximity to water sources. Additionally, upon reaching hibernation sites, they do not enter the water directly to initiate hibernation. Therefore, in subsequent research, providing effective cover for the frogs will be of paramount significance. There are notable differences in the intestinal microbiota between male and female *R. dybowskii*, possibly because female frogs require more energy for the development of reproductive glands. According to the species composition heatmap, 18:00 is the optimal feeding time for *R. dybowskii*. Our study successfully reveals the circadian rhythms of the behavior and gut microbiota in *R. dybowskii* during the migration period. However, it also has limitations. Due to the unique habits and population density of *R. dybowskii*, collecting samples from different seasons and conducting large-scale statistics will have considerable research value in future research. In conclusion, our findings contribute to understanding the circadian rhythm of *R. dybowskii* and providing crucial insights for improving their breeding strategies.

## Figures and Tables

**Table 1:** Basic metrics of *R. dybowskii*.

Age	Sex	Weight (g)	Length (mm)	Sex Ratio
froglets	♀	5.41	39.44	87:138
	♂	4.81	36.75	
adult frogs	♀	24.24	62.41	64:112
	♂	17.68	56.37	

*Note.* (♀) Female, (♂) Male.

## Data Availability

The raw reads have been deposited in the NCBI database (BioSample number SAMN39511903).

## Supplementary Materials

The following supporting information can be downloaded at: https://www.mdpi.com/article/10.3390/life14030322/s1, Figure S1. The gut bacterial community’s relative abundance at the genus level (A,B) and species level (C,D); Figure S2. Venn diagram of distribution of OTUs in intestinal bacteria of Dybowski’s frogs at different time points; Figure S3. Correlation analyses of the gut bacteria. (A,B) Phylum level. (C,D) Species level; Figure S4. Principal coordinate analysis (PCoA) of the gut bacterial community and nonmetric multidimensional scaling of the gut bacterial community; Figure S5. The relative abundance of metabolic pathways; Table S1. Sequencing statistics of gut bacteria; Table S2. Number of ASVs of different taxonomic levels in gut samples.
