# Supplementary material for: The Circadian Rhythm of the Behavior and Gut Microbiota in Dybowski’s Frogs (Rana dybowskii) during the Autumn Migration Period
Source: Life (Basel). 2024 Feb 28;14(3):322. doi: 10.3390/life14030322 (PMC10971735; doi:10.3390/life14030322)
Supplement: Supplementary file 1 [file life-14-00322-s001.zip › Figure S1.pdf]

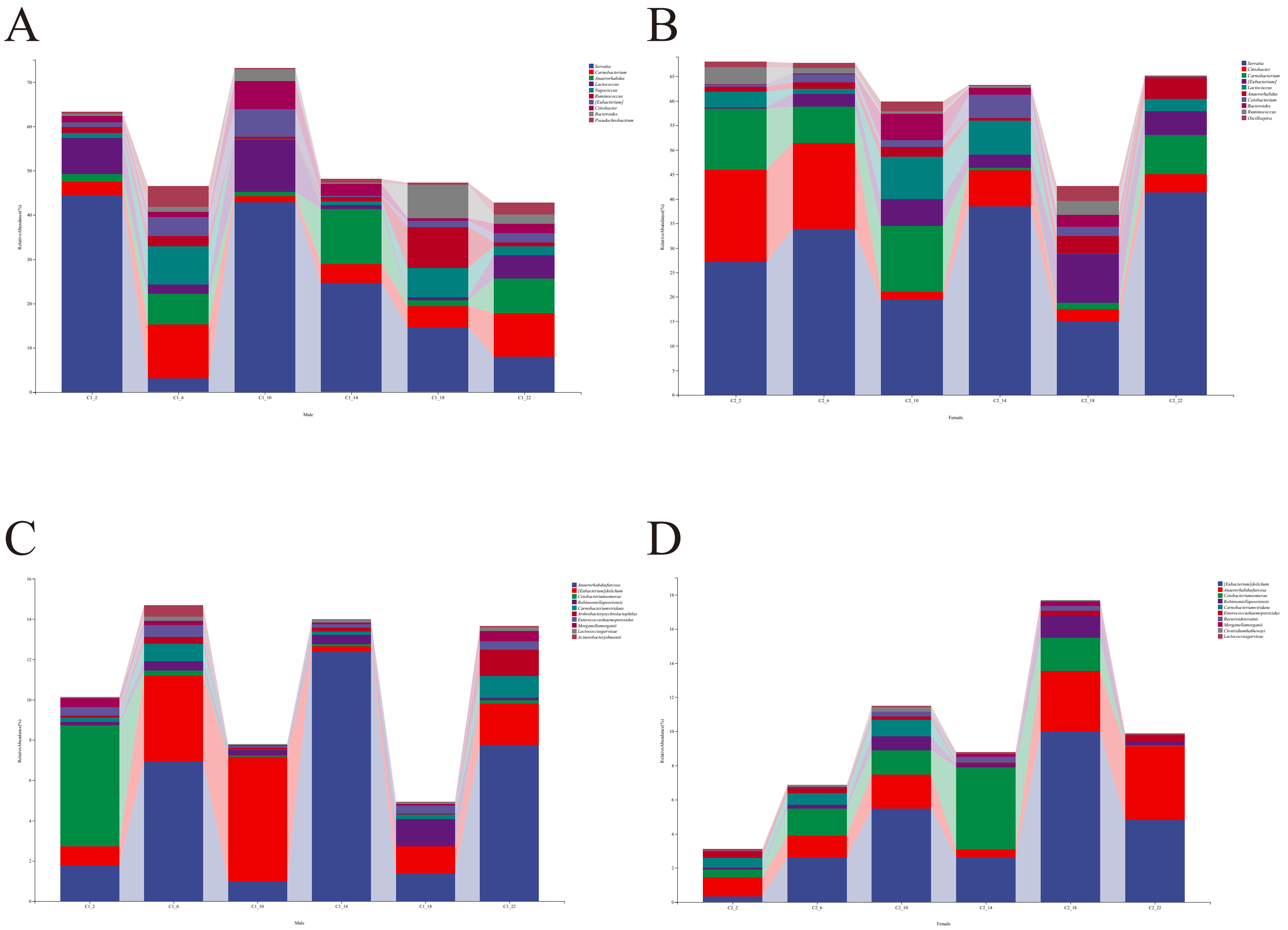
A
B
C
D
Relative Abundance(%)
Male
Female
C1_2
C1_6
C1_10
C1_14
C1_18
C1_22
C2_2
C2_6
C2_10
C2_14
C2_18
C2_22
Serratia
Carnobacterium
Anaerorhabdus
Lactococcus
Vagococcus
Ruminococcus
[Eubacterium]
Citrobacter
Bacteroides
Pseudochrobactrum
Serratia
Citrobacter
Carnobacterium
[Eubacterium]
Lactococcus
Anaerorhabdus
Cetobacterium
Bacteroides
Ruminococcus
Oscillospira
Anaerorhabdusfurcosa
[Eubacterium]dolichum
Cetobacteriumsomerae
Robinsoniellapeoriensis
Carnobacteriumviridans
Arthrobacterpsychrolactophilus
Enterococcushaemoperoxidus
Morganellamorganii
Lactococcusgarvieae
Acinetobacterjohnsonii
[Eubacterium]dolichum
Anaerorhabdusfurcosa
Cetobacteriumsomerae
Robinsoniellapeoriensis
Carnobacteriumviridans
Enterococcushaemoperoxidus
Bacteroidesovatus
Morganellamorganii
Clostridiumhathewayi
Lactococcusgarvieae
