# Supplementary material for: The Circadian Rhythm of the Behavior and Gut Microbiota in Dybowski’s Frogs (Rana dybowskii) during the Autumn Migration Period
Source: Life (Basel). 2024 Feb 28;14(3):322. doi: 10.3390/life14030322 (PMC10971735; doi:10.3390/life14030322)
Supplement: Supplementary file 1 [file life-14-00322-s001.zip › Table S1.pdf]

Table S1 Sequencing statistics of gut bacteria

SampleID	Input	Filtered	Denoised	Merged	Non-chimeric	Non-singleton
C1_2_1	67259	64014	62701	58564	48993	48957
C1_2_2	107964	98454	96470	88661	60117	60053
C1_2_3	99851	92096	90663	86441	71792	71757
C1_6_1	114551	103831	100161	84886	66225	66088
C1_6_2	117108	105817	103271	93920	71269	71205
C1_6_3	117720	105183	102477	89348	50116	49988
C1_10_1	99896	91634	89783	83610	72109	72063
C1_10_2	117652	106861	104826	94595	73251	73170
C1_10_3	62083	59497	58037	52732	42993	42929
C1_14_1	95567	88526	87634	86105	77414	77402
C1_14_2	58088	55899	55312	53709	48620	48607
C1_14_3	96351	88956	87130	81050	68459	68418
C1_18_1	116760	105909	103260	93981	69735	69691
C1_18_2	88337	82279	81395	79412	52687	52673
C1_18_3	62698	59634	58120	51536	38883	38837
C1_22_1	62817	60298	58505	51174	35485	35381
C1_22_2	71253	64443	63299	59337	53188	53170
C1_22_3	64802	61817	60582	56380	37123	37074
C2_2_1	95609	87620	85879	80144	62723	62686
C2_2_2	67177	64521	63674	61529	46306	46288
C2_2_3	72709	69816	68915	66535	56531	56511
C2_6_1	69813	66631	65457	61624	48009	47978
C2_6_2	63627	61283	60148	56835	48297	48269
C2_6_3	66822	64189	63315	60798	45914	45883
C2_10_1	61386	58792	56624	48210	30962	30882
C2_10_2	63693	61057	59884	56219	37190	37171
C2_10_3	66642	63644	62668	59660	46547	46520
C2_14_1	75469	71921	71407	70034	63769	63760
C2_14_2	74395	70542	69881	68186	62627	62615
C2_14_3	64998	61835	60846	58325	47041	47007
C2_18_1	60468	56876	54966	47003	34154	34110
C2_18_2	70924	67540	65137	56829	46048	46007
C2_18_3	79311	75413	73728	69245	55764	55732
C2_22_1	61453	58502	57526	55942	41921	41895
C2_22_2	60788	58191	57277	54937	42328	42321
C2_22_3	68316	64777	63835	62141	53776	53751

Table S2 Number of ASVs of different taxonomic levels in gut samples.

ID	domain	phylum	class	order	family	genus	species
C1_2_1	13	0	2	41	174	197	51
C1_2_2	13	3	5	55	138	194	73
C1_2_3	6	1	4	24	152	171	47
C1_6_1	15	2	2	120	284	347	105
C1_6_2	7	2	2	69	219	297	88
C1_6_3	11	8	6	79	209	291	106
C1_10_1	12	2	3	31	192	215	48
C1_10_2	5	4	1	48	161	170	34
C1_10_3	8	1	5	53	172	180	52
C1_14_1	1	0	3	9	89	122	35
C1_14_2	1	0	2	17	109	143	43
C1_14_3	1	0	3	39	157	154	36
C1_18_1	6	3	2	54	122	188	38
C1_18_2	0	0	6	12	111	146	27
C1_18_3	14	2	1	47	148	161	33
C1_22_1	24	6	8	78	239	339	90
C1_22_2	2	1	0	43	145	216	56
C1_22_3	8	5	9	39	161	159	52
C2_2_1	2	6	4	32	135	182	28
C2_2_2	4	5	6	23	130	129	36
C2_2_3	6	0	3	21	114	120	37
C2_6_1	2	3	4	32	170	169	52
C2_6_2	7	1	1	30	162	130	19
C2_6_3	4	3	6	45	161	166	39
C2_10_1	14	2	4	116	200	221	27
C2_10_2	5	4	7	40	130	162	51
C2_10_3	3	3	1	29	153	152	25
C2_14_1	0	0	2	16	81	73	10
C2_14_2	1	1	1	18	25	60	10
C2_14_3	8	4	2	13	109	95	43
C2_18_1	12	9	4	54	171	148	33
C2_18_2	20	1	4	99	179	220	50
C2_18_3	3	1	0	23	152	162	51
C2_22_1	4	2	0	23	107	115	36
C2_22_2	1	1	2	28	137	91	37
C2_22_3	2	0	4	18	111	113	37
