# Supplementary figures and images for: The Circadian Rhythm of the Behavior and Gut Microbiota in Dybowski’s Frogs (Rana dybowskii) during the Autumn Migration Period
Source: Life (Basel). 2024 Feb 28;14(3):322. doi: 10.3390/life14030322 (PMC10971735; doi:10.3390/life14030322)

# A

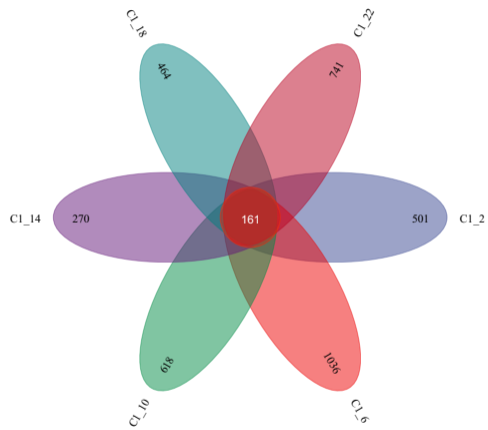

# B

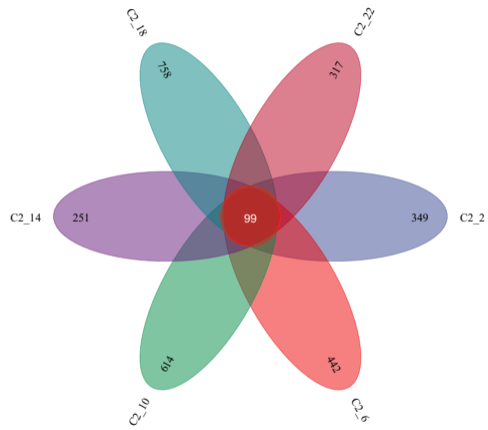

Supplement: Supplementary file 1 [file life-14-00322-s001.zip › Figure S2.pdf]

A

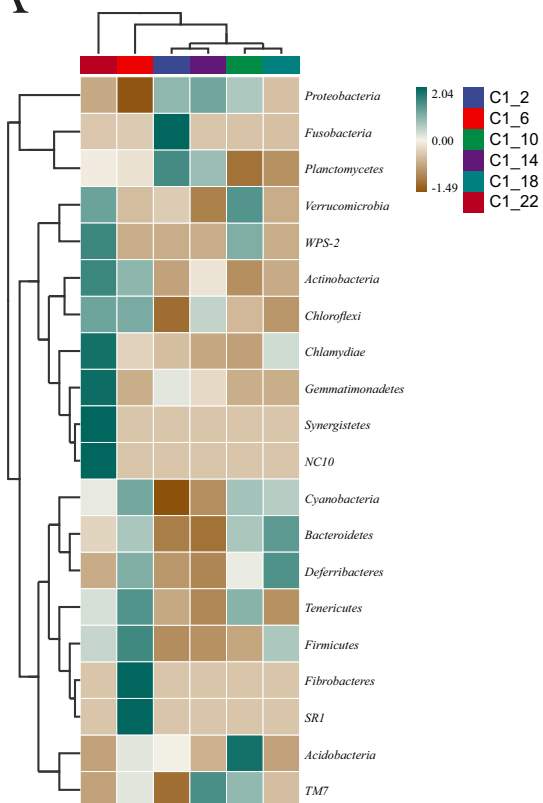

B

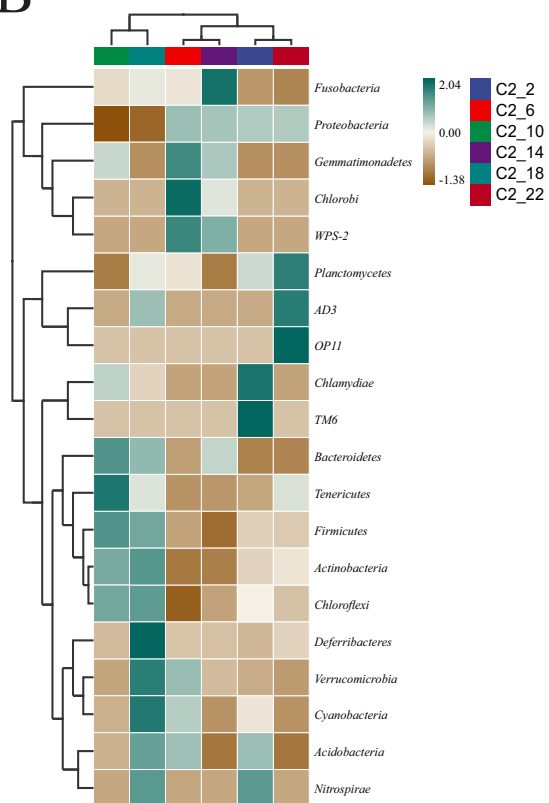

C

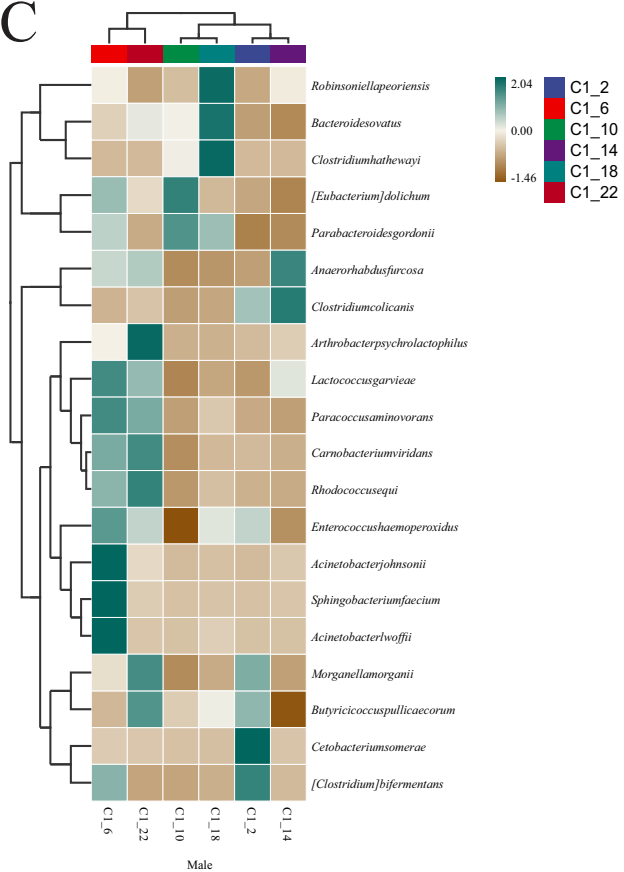

D

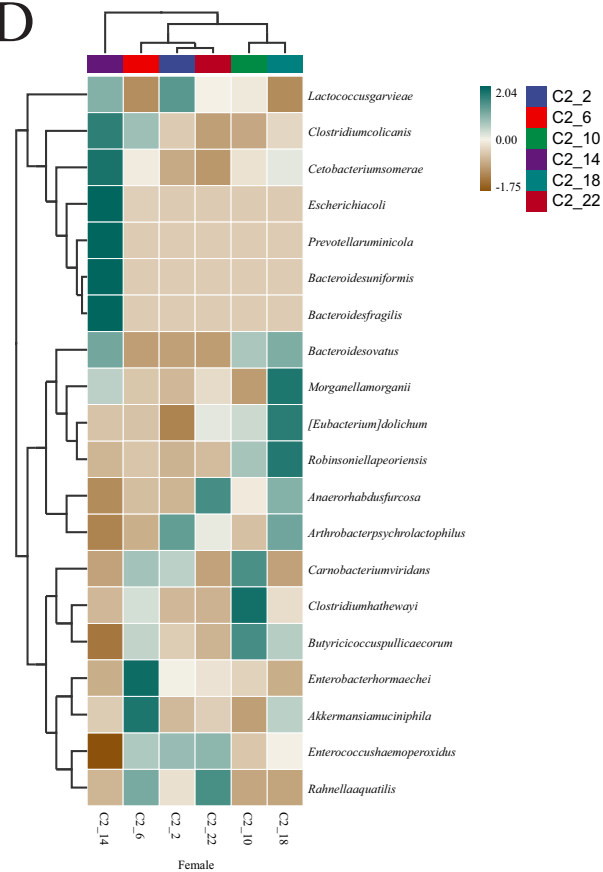

Supplement: Supplementary file 1 [file life-14-00322-s001.zip › Figure S3.pdf]

A

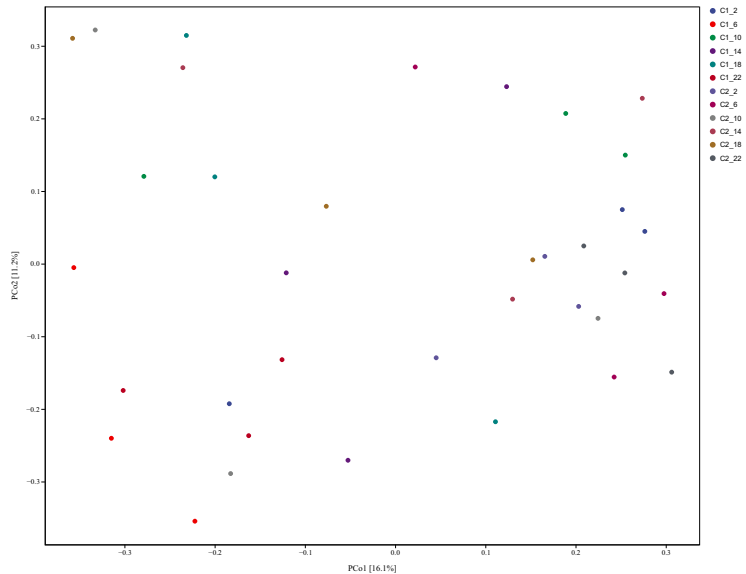

B

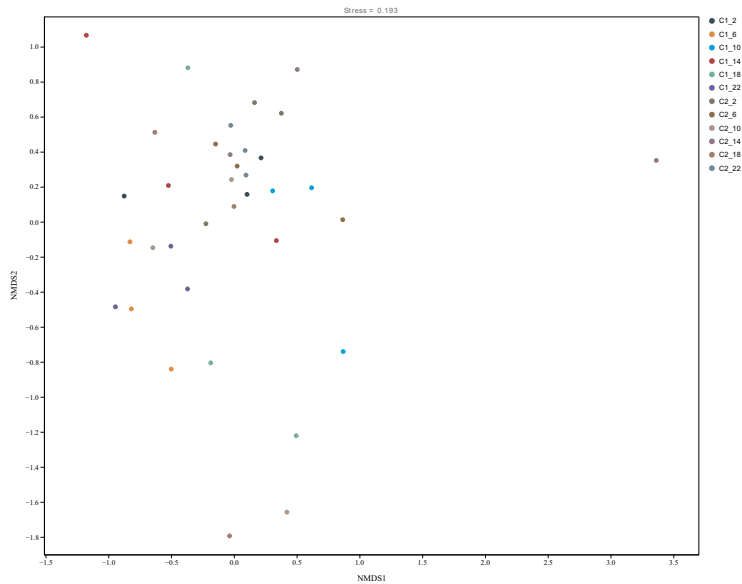

Supplement: Supplementary file 1 [file life-14-00322-s001.zip › Figure S4.pdf]

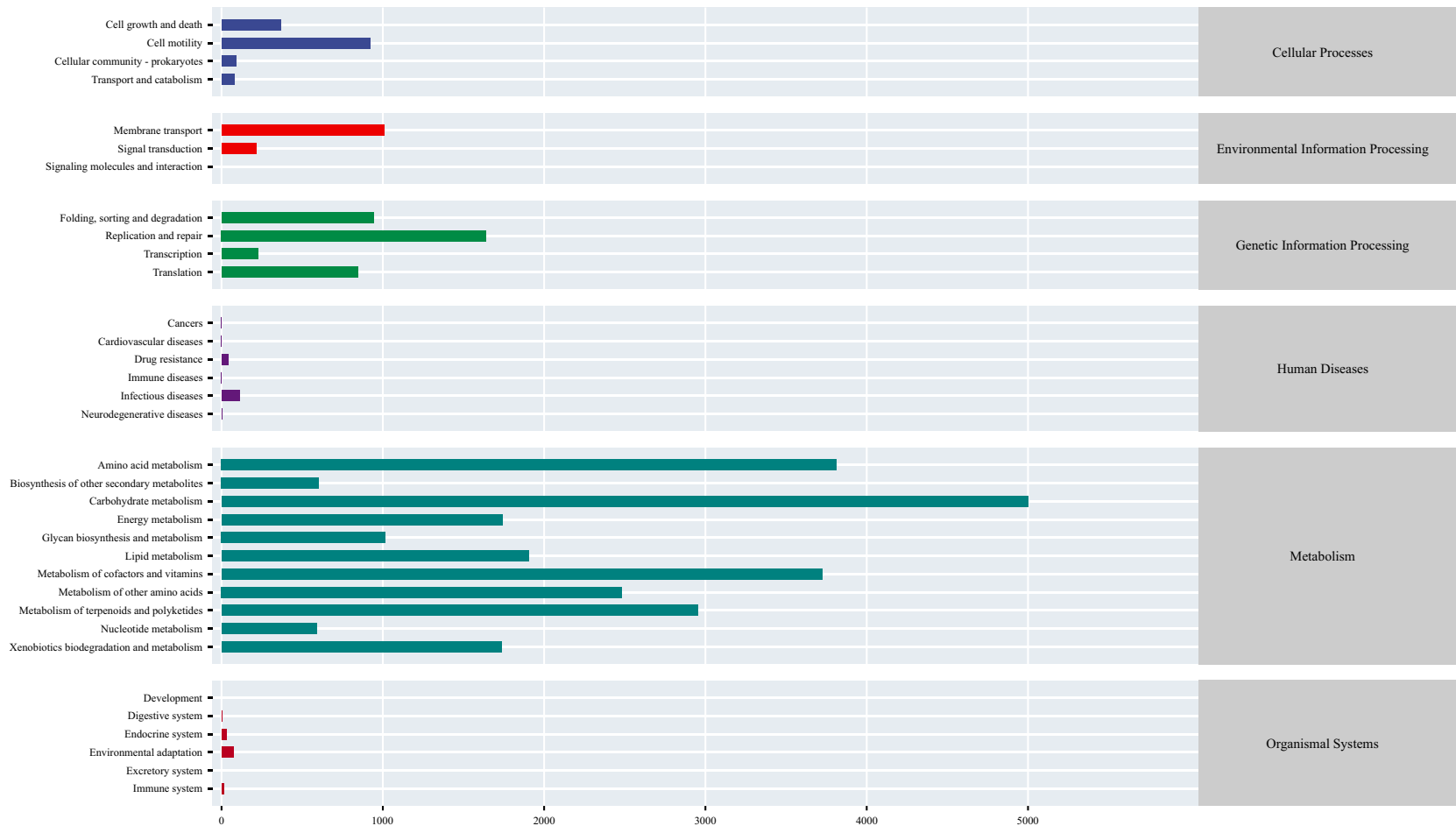

Supplement: Supplementary file 1 [file life-14-00322-s001.zip › Figure S5.pdf]
